# Playing to live: outcome evaluation of a community-based psychosocial expressive arts program for children during the Liberian Ebola epidemic

**DOI:** 10.1017/gmh.2019.1

**Published:** 2019-04-22

**Authors:** C. A Decosimo, J. Hanson, M. Quinn, P. Badu, E. G. Smith

**Affiliations:** 1 Playing to Live, East Tennessee State University, Johnson City, TN, USA; 2 University of Pittsburg, Pittsburgh, PA, USA; 3 East Tennessee State University, Johnson City, TN, USA; 4 Playing to Live, Renewed Energy Serving Humanity, Paynesville, Liberia; 5 Renewed Energy Serving Humanity, Monrovia, Liberia

**Keywords:** Art therapy, Ebola, expressive therapies, Liberia, MHPSS

## Abstract

**Background.:**

This paper reviews the efficacy of a community psychosocial arts program focused on building mental health capacity within post-Ebola Liberia. The aim of this paper was to evaluate the outcome effects of two groups using pre- and post-treatment data. We hypothesized that there would be a difference in symptoms pre- and post-treatment, and the longer program would yield more significant results.

**Methods.:**

There was a total of 870 child participants. Of 40 sites, 24 were selected for a 5-month treatment (TG1) while the remaining 16 sites received 3 months of treatment (TG2). Paired *t* tests and a mixed-model analysis of variance (ANOVA) were used to analyse pre- and post-psychological stress symptoms (PSS) for samples from both groups.

**Results.:**

Separately, treatment group 1 (TG1) and treatment group 2's (TG2) paired *t* test yielded significant results (*p* < 0.001) for the decrease of PSS. The mixed-model ANOVA found that there were significant differences in total pre- and post-test PSS and a significant difference in PSS means over time.

**Conclusions.:**

Results indicated that there was a statistically significant decrease in reported symptoms in both treatment groups pre- to post-intervention and a significant difference in total symptoms over time. However, the findings do not indicate that the longer programming was statistically different compared to the shorter programming. The study presented had gaps in data, largely due to limits in research during the crisis. However, this paper provides a unique case study for challenges that can be faced for project evaluation in emergency settings.

## Background

### Childhood trauma

Early childhood is considered to be a vital development time for one's emotional foundation (Briggs-Gowan *et al*., 2010), and childhood trauma has been found to have an effect on an individual's risk for mental illness, delayed cognitive developmental milestones, substance abuse, relationships and academic issues (Johnson *et al*., 2001; Diehl & Prout, 2002; Wethington *et al*., 2008; Bolton, 2014). The Adverse Childhood Experience study, where childhood trauma was researched through quantifiable measures, found that the higher the score for adverse experiences the higher the risk for chronic obstructive pulmonary disease, smoking, intravenous drug use, and suicide (Felitti & Anda, 2010). A child's environment, especially if they are exposed to multiple elements of adversity, has a large impact on their risk for Post-Traumatic Stress Disorder and trauma-related issues (Berkowitz *et al*., 2011). A community-wide trauma, such as natural disasters, wars, and epidemics, can impart innumerable complications and obstacles for community service agencies and community organizations, which can have a substantial impact and burden on the individuals, families, and communities affected and in need of care (SAMHSA, 2014).

Children who have access to psychosocial support and resources after trauma have a higher potential for recovery and resiliency (Caffo & Belaise, 2003), but in drastically low resource settings, trained mental health clinicians are severely scarce. Additionally, the community and family rarely will prioritize mental health care due to the necessity of basic survival needs. As a result, relying on trained mental health professionals in communities will only address a minuscule fraction of the population in need due to limited trained professionals (Patel *et al*., 2011). In contrast, capacity building focused on training lay therapists has been found to be effective in addressing mental health issues when there is limited access to trained mental health clinicians (Singla *et al*., 2014). Capacity building focuses on working within, identifying, and building on the community's resources and reflects an ecological focus on empowerment, where the community is seen as essential for change. It is best practices to use or incorporate appropriate cultural rituals of healing within mental health and psychosocial programming that embodies a community’s belief system. (IASC, 2007, p. 106–109).

### Liberia's Ebola epidemic and playing to live

From 2013 to 2015, the Ebola Virus Disease (EVD) devastated three countries in West Africa: Liberia, Guinea, and Sierra Leone. By the end of the epidemic, there were over 28 000 cases reported and 11 310 known case-fatalities (WHO Response Team, 2014). This disease not only had a severe physical impact but also had a large and noticeable psychological impact on children and families. During this time, the communities around Monrovia, Liberia were experiencing extreme amounts of stress, loss, and trauma. Prior to the Ebola epidemic, Liberia had a severe gap in available mental health services (Levey *et al*., 2013), and the gap grew significantly larger during the epidemic.

From May–November 2015, with a grant from the United Nations Children's Foundation (UNICEF) and guidance and collaboration with the Liberian Ministry of Health and Social Welfare (MOHSW) and Ministry of Gender (MOGD), the American nonprofit, Playing to Live (PTL), and Liberian-based nonprofit, Renewed Energy Serving Humanity (RESH), a large-scale psychosocial expressive arts program was implemented to address the growing psychosocial and mental health needs of children affected by the epidemic. PTL is an American 501(c)3 organization whose mission is to build effective and sustainable psychosocial community-based expressive art programs targeting the psychosocial needs of children and communities facing mental health issues and trauma. RESH is a Liberian-based nonprofit that focuses on providing psychosocial support to individuals and communities experiencing traumatic events. This paper discusses the quantitative findings of a large-scale project implemented by PTL and RESH, which focused on building mental health and psychosocial support (MHPSS) capacity within the most affected communities. Additionally, there is a discussion on barriers faced in establishing the monitoring and evaluation system, lessons learned, and steps for the future. Decosimo *et al*. (2017) describe the process of development and implementation of this specific program and the qualitative outcomes.

## Methods

### Playing to live program

PTL's clinical team consisted of an art therapist, child life specialist, play therapist, and yoga therapist. The clinical team was tasked to identify best practices in their field and develop a training program for para professionals. The tools and techniques identified for the programming were carefully reviewed, through literature review and consultation with expressive therapy colleagues, to determine what techniques were ethical to teach non-clinicians (Kalmanowitz & Potash, 2010). The aim in bringing these diverse but relational therapeutic professions together was to create a dynamic program that integrated therapeutic expressive arts and life skills. PTL only incorporated theories from the individual expressive art therapy fields in which a professional was active during the program's development.

**Fig. 1.**
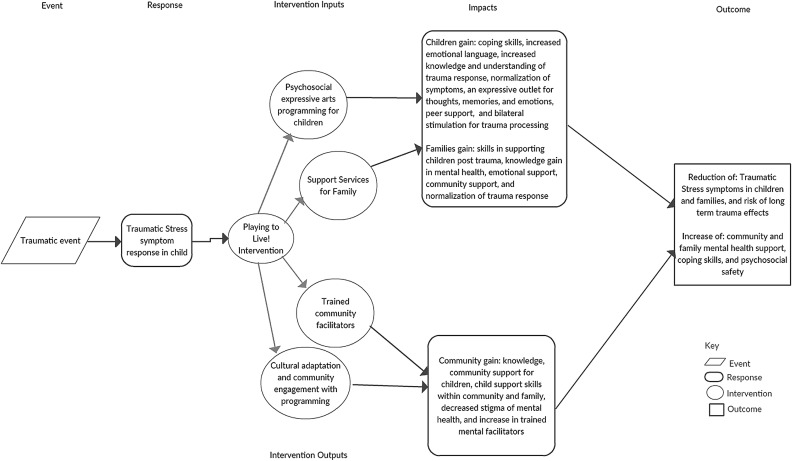
Playing to Live program logic model.

**Fig. 2.**
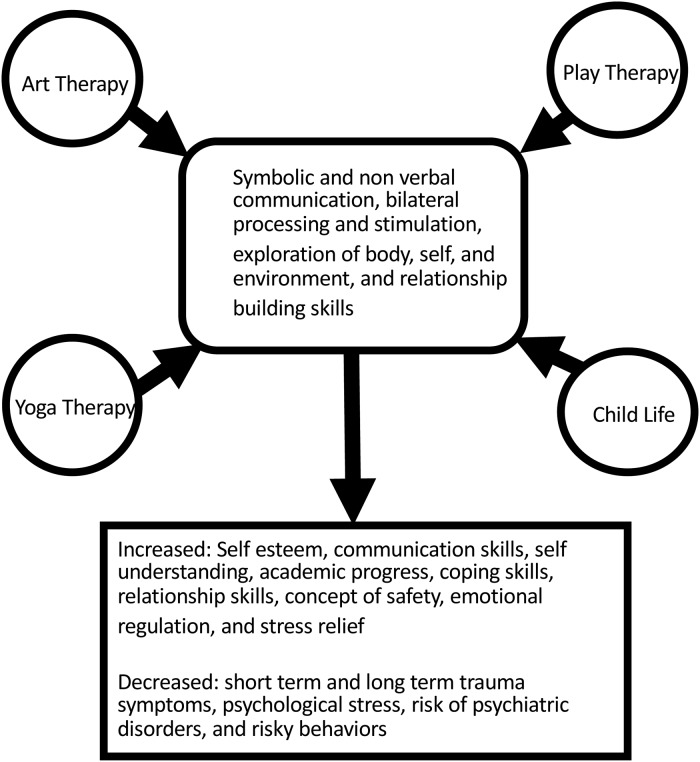
Shared outcomes of expressive art therapy modalities.

The training focused on building healthy relationships, teaching child specific trauma coping skills, and building a safe space for children to express themselves. The program's theory of change was that expressive art activities provide a safe and supportive space for children to experience healing and growth through creativity, mentorship, and peer support. Art and play bypass language barriers and provide room for creative exploration. Figure 1 illustrates a logic model identifying different inputs, outputs, and theorized impacts of the programming. Figure 2 illustrates the shared components of the expressive therapies represented and their projected outcomes, which served as a framework when building this intervention/program. Facilitators were not trained in trauma processing and they were thoroughly taught that facilitating in-depth mental health and trauma work was beyond their training and capacity. RESH assisted in building the program to match Liberian culture and socio-economic needs.

Following two successful pilot programs and consistent guidance and cultural adaptation from Liberian Ministries and leaders, a committee of Liberian leaders and cultural liaisons were gathered to review and comment on the program. The program was adapted based on their recommendations. PTL's team of expressive therapists finalized the multi-day training, follow up training, and 25 expressive arts activities. The training and activities focused on instructing the facilitators on how to conduct activities, support the children beneficiaries, and to communicate with the child's family (Decosimo *et al*., 2017).

The programming and activities for both treatment groups were virtually the same. Facilitators were trained in the activities and instructed to implement at least two to three activities per week. The activities included an explanation on set up and how to introduce and run the activity, recommendations for how to ask questions and facilitate conversation about the activity, and potential issues to look for during the activity. Potential issues included possible triggers, how to work with children who were struggling, and any likely risks to mitigate. The activities were structured so that children (4–11) and youth (12–18) could easily understand and find enjoyment. An example activity is, ‘My positive future.’ This activity instructed children to use art, play, and storytelling to explore what they want for their future. The goal of this activity was to help children and youth to identify positivity within their current situation to help them build hope and goals towards their future (Decosimo *et al*., 2017). Each activity could be used independently and the facilitators had the option of choosing which one they felt was most beneficial for the day.

### Selection of facilitators and structure of programming

The program hired and trained 40 female Ebola survivors to implement PTL in their communities. Furthermore, 40 RESH employees were hired as psychosocial workers, so that one RESH employee would be partnered with each PTL facilitator to collect data, provide supportive talks to the family, and support the PTL programming. Supportive talks were provided by RESH employees to assist with the Ebola crisis; however, data were not collected during those talks.

The program was implemented in two groups using a delayed treatment model. Treatment Group 1 (TG1), which was 60% (*N*  =  533) of the eligible participants, received 5 months of the program. Treatment Group 2 (TG2), which was 40% (*N*  =  337) of participants, received 3 months of programming and began 2 months after TG1 started. This design was implemented to highlight program differences through the variable of time (Table 1). This remained in compliance with the ministry's requests to focus the majority funding on programming so that all children could receive programming in a timely manner.

**Table 1.** Program monthly implementation (X), Monitoring (M), Pre-Test(O_1_), Post-Test (O_2_)

	Months				
Treatment groups	1	2	3	4	5
Treatment group 1	X, O_1_	X, M	X, M	X, M	X, O_2_
Treatment group 2			O_1,_ X	X, M	X, O_2_

The 40 female Ebola survivors were trained by PTL and RESH to facilitate the program to 15–25 children each within their own communities at least 2–3 times per week during the 5-month (TG1) or 3-month (TG2) implementation period. Child participants were selected based on their residency in the PTL facilitator's community and their guardian's consent to have them involved. Children's symptoms were not screened for entry into the program, as the MOHSW wanted the program to be provided based on the child's exposure to EVD and not on psychological stress symptoms (PSS) that may or may not have initially been expressed.

The study compared pre- and post-tests on the total of seven PSS, which were chosen by the MOHSW/MOGD based on items that were culturally and developmentally applicable for children in Liberia and would provide an indication of how the child was adjusting. The PSS were chosen based on existing literature and are further explained in the data collection section. The research hypothesis was that the children in TG1 would show a higher effect of decreased PSS than TG2 due to participating in programming for two extra months.

### Selection of location & participants

There were 870 children (ages 3–18) enrolled in the PTL/RESH project. The project was conducted in 40 sites that were randomly selected from a comprehensive government list of nearly 100 hot-zone communities. In each of the selected sites, there were children who were Ebola-survivors, from Ebola-infected homes, or were living in an Ebola-affected community. The activities were inclusive to all children no matter their Ebola status, with the goal of supporting reintegration and decreasing stigma. Of the 40 sites, 60% (24) were randomly selected for a 5-month treatment while the remaining 16 sites received 3 months of treatment. The sites were randomly selected by the monitoring and evaluation specialist who blindly selected the sites out of a hat.

### Data collection

RESH collected the data, which included child interviews, with parental consent, on seven PSS, parental/guardian interviews on the child's PSS, and observations of household environmental conditions. Data collection was completed with a scorecard that consisted of a yes/no checklist, measuring key symptoms identified by the MOHSW/MOGD as the common symptoms among persons impacted by EVD (Glayweon & Hanson, 2015). Each symptom was identified and approved by the Liberian MOHSW to be used with children. The score card was tested during the pilot and revised for this program. The seven PSS included: withdrawal (Chapman *et al*., 2001), extreme anger (Davidson *et al*., 1997), bedwetting (Beyerlein, 2014), worry/anxiety (Feldman & Vengrober, 2011), poor eating habits (Qureshi *et al*., 2015), violence, and continual sadness (Stallard, 2006). Data were triangulated by asking the child, his/her caregiver, and the PTL facilitator for confirmation of each symptom. The symptoms were collected as yes or no binary variables, and if the triangulation process showed inconsistent reporting on a symptom, the symptom was coded as not being present. The evaluation team made it a priority to follow the Liberian MOHSW/MOGD guidance for the creation and approval of evaluation measures given the emergency context and lack of resources. Poor eating and bed wetting were symptoms associated by communities as relating to EVD. Additional data collected were demographics, guardian marital status, any abuse or negligence, and any reported school issues. Children were categorized in one of three groups depending on their experience with Ebola: survivor, experienced Ebola in the home, or experienced Ebola in the community.

Pre-test data were collected 1–2 weeks prior to the implementation of each program. TG1's pretest data were collected in late June 2014, and the program began shortly thereafter. TG2's pretest data were collected in late August and program implementation began in early September. Post-test data for both treatment groups were collected when both programs finished in mid-late October 2014. Due to resource limitations, communities were randomly selected for post-test data collection, where ten communities from TG1 and six communities from TG2 were randomly selected out of a hat to collect post data. The selection process was completed by a RESH monitoring and evaluation specialist. Since the communities were randomly selected originally and then randomly selected for the post-test, it is expected that the communities chosen are representative of the entire study population.

TG1 had post-test data for 233 participants from the original 533 children. TG2 had 123 participants with post-tests from the original 337 enrollees. Dropout rates were not recorded in the data. The participants without post-tests were excluded from this analysis. The program and data collection were approved by the MOHSW/MOGD. RESH collected and cleaned the data of all identifying information and sent the anonymous data set to the PTL research team for this analysis. The anonymous data received an expedited review and exemption for secondary analysis from the East Tennessee State University Institutional Review Board (IRB). The final article was submitted to the National Ebola Survivors Network of Liberia, where it received formal approval for dissemination from the network's president.

### Statistical analysis

SPSS software was used for the analyses. Descriptive statistics from TG1 & TG2 were analysed to evaluate whether the populations were comparable (Table 2). The outcome effects on PSS were analysed by comparing the means of pre- and post-tests of the two treatment groups, where 0 indicated no symptoms and 7 indicated the child was experiencing all symptoms. A paired *t* test was used to analyse the effects of the two programs individually, and a mixed-model analysis of variance (ANOVA) between the pre-and post-symptom results for TG1 and TG2 was used to determine if there was statistical significance between the effects of the programming for 5 months compared to 3 months.

**Table 2.** Descriptive statistics for TG1, TG2, and total

	TG1	TG2	TG1 & TG2
	*n* = 233	*n* = 123	*n* = 356
	Mean (s.d.)	Mean (s.d.)	Mean (s.d.)
Age	9.90 (3.57)	10.20 (3.87)	9.97 (3.67)
Descriptive stats	*n* (%)	*n* (%)	*n* (%)
Female	147 (63.1%)	73 (59.3%)	220 (63%)
Male	86 (36.9%)	50 (40.7%)	136 (38.2%)
Mother alive	189 (81.1%)	98 (79.7%)	287 (81%)
Father alive	150 (64.4%)	91 (74%)	241 (64%)
Ebola survivor	4 (1.7%)	1 (0.8%)	5 (1.7%)
Parent died of Ebola	81 (34.8%)	32 (26%)	113 (34.8%)
Child experienced Ebola in the family (excluding parent)	31(13.3%)	23 (19%)	54 (13%)
Child experienced Ebola in the community (Excluding family)	119 (51.1%)	67 (54.5%)	186 (51.1%)

## Results

### Descriptive statistics

All communities that received programming were in close proximity to Monrovia, Liberia and reflected similarities in geography and descriptive statistics. Table 2 highlights the description of children represented in the pre-and post-tests. TG1 (*n*  =  233) had a mean age of 9.90 years old (s.d.  =  3.57), with 63.1% female and 36.9% male participants.TG2 (*n*  =  123) had a mean age of 10.20 years old (s.d. 3.87), with 73% female participants. Table 2 also outlines whether the child participants had living parents, along with their Ebola status. Table 3 shows the mean of PSS by Ebola status for TG1 and TG2, and Table 4 compares the mean of PSS by gender. In both tables, post-test total symptoms primarily decreased, with some exceptions. In most cases, TG2 had higher symptom counts, with some symptoms increasing at the post-test. For example, children in TG2 age 5 increased from 0.38 to 0.63 in crying.

**Table 3.** PSS by Ebola status

Ebola status	Crying		Anger		Withdrawn		Worry		Bad eating		Violence		Bedwetting		Total	
	Pre	Post	Pre	Post	Pre	Post	Pre	Post	Pre	Post	Pre	Post	Pre	Post	Pre	Post
TG1 Ebola survivor	0.25	0.25	0.25	0	0.5	0.25	0.5	0.25	0	0	0	0	0	0	1.5	0.75
TG2 Ebola survivor	0.39	1	0.32	0	1	1	1	1	0	0	0	0	1	0	3	3
TG1 parent died of Ebola	0.51	0.25	0.1	0.07	0.21	0.07	0.27	0.12	0.06	0.04	0.14	0.05	0.06	0.04	1.5	0.56
TG2 parent died of Ebola	0.53	0.5	0.34	0.34	0.47	0.47	0.44	0.5	0.69	0.63	0.09	0.13	0.22	0.16	2.45	2.44
TG1 Child experienced Ebola in the family (excluding parent)	0.58	0.1	0.29	.19	0.19	0.03	0.35	0.23	0.13	0.1	0.32	0.13	0.1	0.06	1.55	0.65
TG2 child experienced Ebola in the family (excluding parent)	0.52	0.39	0.22	0.17	0.17	0.17	0.43	0.3	0.65	0.61	0.26	0.22	0.3	0.26	2	1.65
TG1 child experienced Ebola in the community (Excluding family)	0.27	0.22	0.12	0.08	0.22	0.15	0.18	0.13	0.08	0.07	0.11	0.08	0.13	0.07	0.87	0.62
TG2 child experienced Ebola in the community (Excluding family)	0.28	0.21	0.34	0.21	0.34	0.18	0.36	0.21	0.25	0.22	0.1	0.07	0.13	0.13	1.58	1.03

**Table 4.** PSS means and percent with symptom by gender

	Crying		Anger		Withdrawn		Worry		Bad eating		Violence		Bedwetting		Total symptoms	
	Pre	Post	Pre	Post	Pre	Post	Pre	Post	Pre	Post	Pre	Post	Pre	Post	Pre	Post
TG 1 Male mean	0.42	0.20	0.17	0.09	0.21	0.09	0.16	0.06	0.12	0.10	0.17	0.05	0.13	0.08	1.38	0.67
TG1 Male *N*%	36 (40%)	17 (34.0%)	15 (45.5%)	8 (9.3%)	18 (36.0%)	8 (32.0%)	14 (24.6%)	5 (15.2%)	10 (52.6%)	9 (64.3%)	15 (44.1%)	4 (22.2%)	11 (45.8%)	7 (41.2%)		
TG 1 Female means	0.37	0.22	0.12	0.10	0.22	0.12	0.29	0.19	0.06	0.03	0.13	0.10	0.09	0.07	1.28	0.82
TG 1 Female *N*%	54 (60%)	33 (22.4%)	18 (54.5%)	14 (63.6%)	32 (64.0%)	17 (68.0%)	43 (75.4%)	28 (84.8%)	9 (47.4%)	5 (35.7%)	19 (55.9%)	14 (77.8%)	13 (54.2%)	10 (58.8%)		
TG2 Male mean	0.34	0.38	0.28	0.12	0.36	0.20	0.36	0.32	0.48	0.44	0.12	0.12	0.18	0.22	1.82	1.46
TG2 Male *N*%	17 (34.7%)	19 (47.5%)	14 (35.9%)	6 (20.7%)	18 (42.9%)	10 (32.3%)	18 (37.5%)	16 (43.2%)	24 (43.6%)	22 (44.9%)	6 (37.5%)	6 (42.9%)	9 (39.1%)	11 (55.0%)		
TG 2 Female mean	0.40	0.29	0.32	0.32	0.34	0.29	0.39	0.29	0.45	0.37	0.13	0.11	0.19	0.12	1.89	1.55
TG 2 Female *N*%	32 (65.3%)	21 (52.5%)	25 (64.1%)	23 (79.3%)	24 (57.1%)	21 (67.7%)	30 (62.5%)	21 (56.8%)	31 (56.4%)	27 (55.1%)	10 (62.5%)	8 (57.1%)	14 (60.9%)	9 (45.0%)		

### Paired *t* tests

Paired *t* tests were completed for each treatment group separately to analyse mean differences in PSS scores (range 0–7) pre- to post-intervention. Table 5 outlines the paired sample statistics of TG1's 5-month implementation. TG1's total pre-treatment PSS mean was 1.32 (s.d. = 0.93), with a post-treatment PSS mean of 0.77 (s.d.  =  0.73).

**Table 5.** Pre to Post- Intervention PSS^a^ Mean Scores and Paired Sample *t* test for TG1 and TG2

	PSS Mean Scores		Paired Differences					
	Mean (s.d.)	s.e. Mean	Mean (s.d.)	s.e. Mean	95% CI	t	df	*p* value
TG 1 Baseline PSS	1.32 (0.93)	0.06	0.55 (0.96)	0.06	0.43, 0.67	8.74	232	*p* < 0.001
TG1 Post-intervention PSS	0.77 (0.73)	0.05						
TG2 Baseline PSS	2.22 (1.45)	0.13	0.43 (1.19)	0.11	0.21, 0.64	3.93	122	*p* < 0.001
TG2 Post-intervention PSS	1.79 (1.39)	0.13						

a PSS- Psychological Stress Symptoms. The score ranged from 0 to 7.

*Note:* Significant at the *p* < 0.001

Table 5 outlines the paired sample statistics of the TG2's 3-month implementation. TG2's pre-treatment mean was 2.22 (s.d.  =  1.45), and the post-treatment mean was 1.79 (s.d. = 1.392). Table 5 shows the paired-samples-*t* test, which was performed to test the significance between the two assessments. Table 5 shows a significant decrease of total symptoms for both TG1 [(mean difference = 0.55, s.d.  =  0.96); *t*_(232)_ = 8.74, *p* < 0.001)] and TG2 [(mean difference = 0.43, s.d.  =  1.19); *t*_(122)_ = 3.93, *p* < 0.001)] after the implementation of PTL.

### Mixed model ANOVA

The ANOVA test showed that there was a significant difference between the two treatment groups on total symptoms pre- to post-program, *F*_(1,354)_ = 69.46, *p* < 0.001 (Table 6). This indicates that TG2 had significantly higher symptoms of pre- and post-intervention. Additionally, there was a significant difference in total symptoms over time (*F*_(1_,_354)_  =  84.33, *p* < 0.001) (Table 6). However, the interaction between treatment groups and time was not significant (*p*  =  0.28).

**Table 6.** ANOVA tests

Measure:	Time					
		Type III sum of		Mean		
Source		Squares	df	Square	*F*	Sig.
Within-subjects contrasts in TG1 compared to TG2						
Time	Linear	38.04	1	38.1	69.46	*p* < 0.001
Time × Treatment Groups	Linear	0.65	1	0.65	1.18	0.28
Error (factor 1)	Linear	193.85	354	0.55		
Between-subjects effects in TG1 compared to TG2						
Treatment groups		147.49	1	147.48	84.33	*p* < 0.001
Error		619.14	354	1.75		

## Discussion

### Discussion of analysis

There was a statistically significant decrease in reported symptoms in both treatment groups pre- to post-intervention and a significant difference in total symptoms over time. However, while those in TG1 and TG2 had different scores and there were changes in PSS scores across time points, the findings do not indicate that the longer programming was statistically different compared to the shorter programming. This finding does not provide a comprehensive indication that longer programming was more beneficial, but it does support the growing evidence that expressive art activities have short-term effects on the improvement of physical and mental health and social wellbeing (Morris *et al*., 2014).

However, it should be noted that TG2 had a higher mean at pre-test of total symptoms than did TG1. TG2's pre-data were collected 2 months after TG1's pre-data, which indicates that PSS increased over 2 months after the end of the epidemic. It is unclear from the interview of key informants during the implementation time, whether a community-wide event could have impacted TG2's stress symptoms over the 2-month period, though since TG1's symptoms decreased over the 5-month time, it seems unlikely that an additional community-wide event caused increased stress symptoms. The demographics of both treatment groups were similar, and since selection of communities for the two treatment groups was randomized, this difference in the two pre-test symptoms suggests that symptoms rose due to not receiving mental health support immediately after the trauma, which could infer PTL programming acted as a barrier to further development of stress symptoms for TG1. It also highlights the need for sustainable programming for children who have experienced a traumatic event. Children's reactions to trauma are not immediate; at times they can appear weeks or months after the trauma event (Wethington *et al*., 2008). A child might not immediately show signs of traumatic stress, which could lessen their chance of receiving mental health care and support, but these data suggest symptoms could increase over time.

### Ethical considerations

During the process of piloting and implementing this program, PTL and RESH followed the Liberian Ministry's ethical and cultural guidance. Due to limited resources and the crisis at hand, PTL was instructed to focus on program implementation *v.* using monetary and personnel resources for researching the program. From this guidance, PTL & RESH built a monitoring and evaluation process to capture the outcomes of the programming as best as possible given the limited resources available.

Despite those limitations, a strength in the data collection was the triangulation of data. The RESH and PTL employees were trained in culturally appropriate techniques of assessing and inquiring about PSS. By asking the child, the caregiver, and assessing through observation validity was enhanced in the decision to confirm or deny symptoms. A symptom was not recorded as a yes unless all three sources confirmed this to be true.

Ethical considerations were taken into consideration when deciding on the timeline to collect data for the delayed treatment group, and it was decided by the partnership and Liberian guidance that it would be too difficult to collect data on the delayed group 2 months prior to actual implementation. This was to avoid the children becoming prematurely excited for programming.

### Limitations

There were some key limitations to the data collection and analysis. First, baseline data collection included case files that had missing or inaccurate information, mostly explained by the challenge of collecting data within weeks of the Ebola crisis ending. Social and cultural effects from the crisis provided great challenges to the data collection team, and incomplete data were removed from this study. Some of these challenges included children moving away and fears of being infected if they engaged in community activities. Secondly, 510 participants were not included in this analysis because they were not assessed for post data collection. Due to limitations in data collection resources, the evaluation team randomly selected post-test recipients to represent the program and not all participants had post-test data collected. Despite randomization, there are still gaps in the research framework that could have missed cluster effects occurring in specific communities.

Additionally, due to the absence of a midpoint assessment of stress symptoms and data for TG2 not being collected at the same time as TG1, this design is not a true delayed treatment design. The absence of having a control group creates gaps in knowledge and true reliability of the findings. A formal delayed treatment framework would include a pre, mid, and post evaluation structure, in which two nonequivalent groups received the treatment at different times and in a delayed sequence. This program design did not have the budget for a midpoint evaluation. This study analysed the two treatment groups as separate treatments to account for these limitations and to accommodate the recommendations and requests of the Liberian ministries. However, a true delayed treatment design would provide a stronger evaluation of the progression of two treatment groups.

All of these limitations demonstrate the obstacles often faced by in-field evaluation teams in mental and psychosocial programming in developing countries, regularly compounded by high-risk, post-disaster settings. This project team worked closely with government and implementation partners to find solutions to these unanticipated challenges as they arose on the ground. Data collectors were operating in sites heavily affected by Ebola, and still under threat of another outbreak. Their dedication and bravery to establishing a baseline pretest so near the epicenter of the crisis is commended.

To truly understand the value of a program, it is essential to analyse if a program or project has a lasting effect and is sustained over time (Ager *et al*., 2011). These post data were collected directly after the program implementation due to limitations in resources. An ideal model for moving forward is to seek resources to evaluate the long-term impact of psychosocial programs, which would include multiple post data collections over time. This should include the effects on the individuals, families, and surrounding communities. For a program like PTL in Liberia, it would be important to evaluate the child participant's trauma symptoms over a longer period of time. This would build a more in-depth understanding of post-trauma mental health programming and its impact, and, as stated before, a comparison or control group would aid in deciphering how the program influenced the child's stress symptoms compared to other factors. It should be noted, that a PTL representative did visit a few of the communities included in this program a year after the program ended. While data were not able to be collected at this time since the program was finished, it was helpful to see that the former PTL facilitators/ Ebola survivors were still using the activities voluntarily with community children and with their own children. This observation supports PTL's theory that by focusing on building skills within the community, through culturally adapted training and easy to use activities, a sustainable change can be made.

### Recommendations

There is a paucity of research on long-term art therapy programming in emergency settings (Orr, 2007), though art therapy research highlights a significant effect on behaviors and trauma symptoms when individuals receive long-term care (Siayton *et al*., 2010). It is our recommendation that a long-term evaluation framework is created for future programs, with a focus on researching lasting impacts.

A recommendation for future work is to establish an analytic and behavioral framework for similar programming prior to a crisis response. This implementation framework can be used as a theoretically-based foundation for program development and implementation. The framework should include essential steps needed prior to implementation that inform cultural significance and needs of the community, involvement of stakeholders, and how the elements of the program will influence outcomes. Additionally, program developers and partnered organizations should ensure that all appropriate ethics and community leadership are involved, consulted, and in agreement with the program implementation and evaluation plan. Many factors were faced by the local implementation team due to the public health emergency context and the multi-agency request for a timely implementation, therefore accessing and submitting to the local IRB was not achieved. In place of a formal IRB, the local team formed a group of representatives from the Liberian ministries to guide and oversee the development, monitoring and evaluation, and implementation processes. The authors of this paper later submitted the secondary data set for expedited IRB approval.

Secondly, a framework for monitoring and evaluation and outcome measurements is also recommended. This study followed the recommendations from the Liberian ministries and focused on stress symptoms, where the data validity was strengthened by utilizing a triangulation technique that involved collecting data on seven potential stress symptoms from the child, caregiver, and facilitator/social worker. Additional measures on behavior, attitudes, knowledge gain, and perceptions would further inform the understanding of the program's outcome. The development of impact models specific to the different aspects of programming would provide a more in-depth understanding of how they influence change. This could include how therapeutic expressive arts impact childhood trauma symptoms, the impact of community support for children, the impact of community-wide knowledge gain on trauma and mental health, and the impact of psychosocial skill development among local leaders.

## Conclusion

This study highlights the impact a community-based psychosocial expressive arts program had on a child's stress response to the EVD outbreak. Support programming significantly reduced PSS symptoms post-program. This clinical implication suggests the urgent need for psychosocial support programming after a trauma. It is important to note that resources for data collection and program development can be severely limited during a crisis, and it is essential to follow the leadership of the local government and entities. Using low-cost techniques like triangulation during data collection can be an effective tool for building validity and the development of programming and evaluation frameworks prior to an emergency will provide a more thorough and efficient approach. Evaluation frameworks that include analytic and behavioral elements and impact models will provide a stronger understanding of how each component of development, programming, and implementation influence outcome effects.

While further research is needed to show a more in-depth picture of the impact of expressive art therapy techniques in emergency settings like the Ebola epidemic, a growing number of reports are showing the effects of expressive art therapy on reducing trauma stress symptoms (Orr, 2007). The theory states that art and expression provide a portal for children to give meaning to confusion and fear. The sensory aspect of art and play provides a space for a child to explore relationships, symbolism, and emotions (Byers, 1996). The child participants in the PTL programming were provided a safe and supportive space to explore and communicate their experiences from the epidemic. The art and play provided the children a modality to connect through symbolism and metaphors, allowing them to take control of their chaotic environment. The results from this analysis suggest that PTL programming could have acted as a protective factor towards the development of further trauma stress symptoms from the children of TG1.

For future programming, the development of impact models specific to the different aspects of PTL's program will provide a more in-depth understanding of how they influence change. The different aspects include how therapeutic expressive arts impact childhood trauma symptoms, the impact of community support for children, the impact of community-wide knowledge gain on trauma and mental health, and the impact of psychosocial skill development among local leaders. Qualitative data and process evaluations are also a key component of a thorough evaluation. Decosimo *et al*. (2017) explains the qualitative assessment of this specific program and highlights the process taken to implement a program during the Ebola epidemic.

This evaluation builds a foundation for future investigation of therapeutic expressive arts programming and provides a key case study reflecting on potential evaluation obstacles to overcome when providing psychosocial programming in post-epidemic settings. Despite limitations in methodology due to issues with data collection and completeness, this study provides worthwhile information regarding children's PSS in the midst of a major infectious disease epidemic, which is unprecedented in the literature. Further, the study provides an impactful approach on how to successfully navigate implementation of a program to assist with long-term effects of an epidemic that caused high-levels of mortality in an already low-resource environment.

## References

[ref1] Ager A, Ager W, Stavrou V, Boothby N (2011). Inter-Agency Guide to the Evaluation of Psychosocial Programming in Emergencies. UNICEF: New York. p.16.

[ref4] Berkowitz SJ, Stover CS, Marans SR (2011). The child and family traumatic stress intervention: secondary prevention for youth at risk of developing PTSD. Journal of Child Psychology and Psychiatry 52, 676–685. (10.1111/j.1469-7610.2010.02321.x). 20868370 PMC3096712

[ref6] Beyerlein BA (2014). Need for trauma-informed care within the foster care system: a policy issue. Child Welfare 93, 7–22.26030986

[ref7] Bolton PA (2014). The unknown role of mental health in global development. The Yale Journal of Biology and Medicine 87, 241–249. (http://www.pubmedcentral.nih.gov/articlerender.fcgi?artid?=?4144279&tool?=?pmcentrez&rendertype?=?abstract). 25191140 PMC4144279

[ref8] Briggs-Gowan MJ, Carter AS, Clark R, Augustyn M, McCarthy KJ, Ford JD (2010). Exposure to potentially traumatic events in early childhood: differential links to emergent psychopathology. Journal of Child Psychology and Psychiatry and Allied Disciplines 51, 1132–1140. (10.1111/j.1469-7610.2010.02256.x). PMC3106304 20840502

[ref9] Byers JG (1996). Children of the stones: art therapy interventions in the West Bank. Art Therapy 13, 238–243. (10.1080/07421656.1996.10759231).

[ref10] Caffo E, Belaise C (2003). Psychological aspects of traumatic injury in children and adolescents. Child Adolescent. Psychiatry. Clinic. North America 12, 493–535. 10.1016/s1056-4993(03)00004-x 12910820

[ref12] Chapman L, Morabito D, Ladakakos C, Schreier H, Knudson MM (2001). The effectiveness of art therapy interventions in reducing Post Traumatic Stress Disorder (PTSD) symptoms in pediatric trauma patients. Art Therapy 18, 100–104. (10.1080/07421656.2001.10129750).

[ref14] Davidson JR, Book SW, Colket JT, Tupler LA, Roth S, David D, Feldman ME (1997). Assessment of a new self-rating scale for post-traumatic stress disorder. Psychological Medicine 27, 153–160. (10.1017/S0033291796004229). 9122295

[ref15] Decosimo CA, Hanson JE, Boland CR, Slawson D, Littleton MA, Quinn M (2017). A process description of playing to live! A community psychosocial arts program during Ebola. Journal of Social, Behavioral, and Health Sciences 11, 176–199. (10.5590/JSBHS.2017.11.1.12).

[ref16] Diehl AS, Prout MF (2002). Effects of posttraumatic stress disorder and child sexual abuse on self-efficacy development. American Journal of Orthopsychiatry 72, 262–265. (10.1037//0002-9432.72.2.262). 15792065

[ref20] Feldman R, Vengrober A (2011). Posttraumatic stress disorder in infants and young children exposed to war-related trauma. Journal of the American Academy of Child and Adolescent Psychiatry 50, 645–658. (10.1016/j.jaac.2011.03.001). 21703492

[ref22] Felitti VJ, Anda RF (2010). The relationship of adverse childhood experiences to adult health status. Kaiser Permanente and the Center for Disease Control 48, 1–14.

[ref25] Glayweon M, Hanson J (2015). Survivors’ network survey summary report. Ebola Survivors’ Network of the Ministry of Health-Liberia, Government of Liberia.

[ref40] Inter-Agency Standing Committee (IASC). (2007). IASC guidelines on mental health and psychosocial support in emergency settings. IASC: Geneva. 10.1080/09540261.2022.2147420 36502397

[ref29] Johnson DM, Pike JL, Chard KM (2001). Factors predicting PTSD, depression, and dissociative severity in female treatment-seeking childhood sexual abuse survivors. Child Abuse and Neglect 25, 179–198. (10.1016/S0145-2134(00)00225-8). 11214810

[ref30] Kalmanowitz D, Potash JS (2010). Ethical considerations in the global teaching and promotion of art therapy to non-art therapists. Arts in Psychotherapy 37, 20–26. (10.1016/j.aip.2009.11.002).

[ref32] Levey E, Borba C, Harris B, Carney J, Domínguez S, Wang E, Henderson D (2013). Assessment of the needs of vulnerable youth populations in post-conflict Liberia. African Journal of Psychiatry 16, 349–355. (10.4314/ajpsy.v16i5.47). 24051668

[ref35] Morris JH, Kelly C, Toma M, Kroll T, Joice S, Mead G, Donnan P, Williams B (2014). Feasibility study of the effects of art as a creative engagement intervention during stroke rehabilitation on improvement of psychosocial outcomes: study protocol for a single blind randomized controlled trial: the ACES study. Trials 15, 1–12. (10.1186/1745-6215-15-380). 25262168 PMC4190489

[ref37] Orr PP (2007). Art therapy with children after a disaster: a content analysis. The Arts in Psychotherapy 34, 350–361. (10.1016/j.aip.2007.07.002).

[ref38] Patel V, Chowdhary N, Rahman A, Verdeli H (2011). Improving access to psychological treatments: lessons from developing countries. Behaviour Research and Therapy 49, 523–528. (10.1016/j.brat.2011.06.012). 21788012 PMC3242164

[ref41] Qureshi AI, Chughtai M, Loua TO, Pe Kolie J, Camara HFS, Ishfaq MF, N'Dour CT, Beavogui K (2015). Study of Ebola virus disease survivors in Guinea. Clinical Infectious Diseases 61, 1035–1042. (10.1093/cid/civ453). 26060289

[ref43] Siayton SC, Archer JD, Kaplan F (2010). Outcome studies on the efficacy of art therapy: a review of findings. Art Therapy 27, 108–119. (10.1080/07421656.2010.10129660).

[ref44] Singla DR, Weobong B, Nadkarni A, Chowdhary N, Shinde S, Anand A, Fairburn CG, Dimijdan S, Velleman R, Weiss H, Patel V (2014). Improving the scalability of psychological treatments in developing countries: an evaluation of peer-led therapy quality assessment in Goa, India. Behaviour Research and Therapy 60, 53–59. (10.1016/j.brat.2014.06.006). 25064211 PMC4148587

[ref45] Stallard P (2006). Psychological interventions for post-traumatic reactions in children and young people: a review of randomised controlled trials. Clinical Psychology Review 26, 895–911. (10.1016/j.cpr.2005.09.005). 16481081

[ref46] Substance Abuse and Mental Health Services Administration. (2014) *SAMHSA's Concept of Trauma and Guidance for A Trauma-Informed Approach*. HHS Publication No. (SMA) 14-4884 Substance Abuse and Mental Health Services Administration: Rockville, MD.

[ref49] Wethington HR, Hahn RA, Fuqua-Whitley DS, Sipe TA, Crosby AE, Johnson RL, Liberman AM, Mościcki E, Price LN, Tuma FK, Kalra G, Chattopadhyay SK (2008). The effectiveness of interventions to reduce psychological harm from traumatic events among children and adolescents. American Journal of Preventive Medicine 35, 287–313. (10.1016/j.amepre.2008.06.024). 18692745

[ref50] World Health Organization Ebola Response Team. (2014). Ebola virus disease in West Africa – The first 9 months of the epidemic and forward projections. New England Journal of Medicine 371, 1481–1495. 25244186 10.1056/NEJMoa1411100 PMC4235004

